# Black Soldier Fly Can Safely Co-Convert Antibiotic Fermentation Residue and Potato Peel Waste into a Valuable Feed Resource

**DOI:** 10.3390/insects17060550

**Published:** 2026-05-25

**Authors:** Xiaopeng Zhang, Lu Zhao, Gaojie Yu, Ahmed R. Henawy, Longyu Zheng, Feng Huang, Minmin Cai, Ziniu Yu, Jibin Zhang

**Affiliations:** 1 National Key Laboratory of Agricultural Microbiology, Huazhong Agricultural University, Wuhan 430070, Chinaahmed.ragab1@agr.cu.edu.eg (A.R.H.);; 2 College of Life Science and Technology, Huazhong Agricultural University, Wuhan 430070, China; 3 National Engineering Research Center of Microbial Pesticides, Huazhong Agricultural University, Wuhan 430070, China; 4 Gansu Heshengkang Biotechnology Co., Ltd., Lanzhou 733000, China; 5 Gansu Huirui Fermentation Technology Research Institute Co., Ltd., Lanzhou 733000, China; 6 Department of Microbiology, Faculty of Agriculture, Cairo University, Giza 12613, Egypt

**Keywords:** antibiotic fermentation residue, nosiheptide, potato peel waste, black soldier fly larvae, degradation, conversion

## Abstract

Among diverse industrial wastes, antibiotic fermentation residues containing high concentrations of nosiheptide pose significant environmental and health risks. This study demonstrates that black soldier fly larvae (BSFL) can effectively degrade the nosiheptide residues within this fermentation matrix when blended with potato peel waste. Optimal degradation efficiency was achieved at a dry weight ratio of 3:5 (antibiotic fermentation residue to potato peel waste), yielding a 40.02% material reduction, an 8.63% bioconversion rate, and a 55.74% nosiheptide degradation rate. Further optimization of the larva-to-feed ratio enhanced nosiheptide degradation to 58.21%. Following 48 h of gut emptying period, no detectable nosiheptide remained within the tissues of the treated BSFL. The harvested larvae demonstrated high nutritional value, with crude protein and crude fat contents reaching up to 35.64% and 32.65%, respectively. The larvae also contained a comprehensive profile of essential amino acids, with the glutamic acid content exceeding 3%, which enhances feed palatability. Highly concentrated antibiotic treatments significantly increased the relative abundance of Bacteroidetes within the BSFL gut microbiota, with *Dysgonomonas* emerging as the dominant genus. This study highlights a novel strategy for degrading residual nosiheptide and converting waste into a valuable protein source, offering an eco-friendly solution for industrial waste management.

## Simple Summary

This study provides a potential solution for the recycling and utilization of potato peel waste and antibiotic fermentation residues. The black soldier fly larvae with intestinal microorganisms are confirmed to efficiently convert organic waste into insect protein while reducing antibiotic concentrations. In addition, the degradation rate of antibiotics and the conversion efficiency are further enhanced by optimizing the ratio of waste and the ratio of larvae to feed. All of these lay a foundation for this novel strategy for the bio-conversion of potato peel waste and antibiotic fermentation residues.

## 1. Introduction

The advent and subsequent proliferation of antibiotics have fundamentally reshaped the landscapes of clinical medicine and intensive animal husbandry [1]. Among these agents, nosiheptide, a sulfur-rich thiopeptide antibiotic, demonstrates exceptional potency against diverse Gram-positive pathogens [2,3]. Nosiheptide enhances daily weight gain and feed efficiency at lower inclusion levels than competing antibiotics, while its rapid elimination from animal tissues minimizes residue concerns [4]. However, nosiheptide fermentation residue, which consists of leftover biomass from antibiotic production and downstream purification, is a nutrient-rich but risk-laden sludge. This matrix contains inactivated hyphae, spent culture medium, microbial metabolites, process chemicals (coagulants, filter aids) and, importantly, residual bioactive antibiotic. Incomplete extraction results in residual antimicrobial concentrations high enough to facilitate the selection of resistant microorganisms and the horizontal transfer of resistance genes. Without rigorous containment and treatment, this residue serves as a persistent pollution source, potentially disseminating antibiotics and antibiotic resistance genes (ARGs) into soil, groundwater, and food webs [5].

Following the 2008 reclassification of nosiheptide fermentation residue as hazardous waste by Chinese regulators, its management has become a critical environmental priority under China’s dual-carbon goals. Antibiotic elimination primarily proceeds via biodegradation and non-biodegradative pathways, including hydrolysis, photolysis, and oxidative/reductive cleavage [6,7,8]. While traditional disposal methods such as incineration and landfilling are employed, they entail high costs, significant pollution risks, and a lack of resource recovery [9]. Due to the complex and stable molecular structure of nosiheptide, efficient degradation remains challenging; complete structural degradation currently requires high-temperature pyrolysis (>300 °C) or advanced oxidation processes.

Recent research underscores the significant potential of black soldier fly larvae (BSFL) for antibiotic degradation. For instance, the BSFL gut possesses specialized enzymes capable of breaking down the four fused benzene rings of tetracycline; consequently, larvae can degrade tetracycline 1.6 times faster than natural processes [10] and effectively utilize tetracycline fermentation residues [11]. Furthermore, BSFL can degrade ciprofloxacin through quinoline ring hydroxylation, reactive oxygen species (ROS)-mediated ring cleavage, or epoxidation and dehydration of the piperazine moiety. Despite these advancements, the efficacy of BSFL in processing nosiheptide fermentation residue and the specific mechanisms of nosiheptide degradation remain poorly understood. Therefore, this study evaluates BSFL as a dual-purpose system for the bio-remediation of nosiheptide-rich residues and the production of sustainable insect biomass.

Potatoes represent a global staple with annual production exceeding 350 million tons, of which China contributes approximately one-third [12,13]. The industrial processing of this volume generates substantial potato peel waste (PPW), which is frequently landfilled—a practice that facilitates anaerobic decomposition, greenhouse gas emissions, and the leaching of hazardous compounds [14]. Although PPW serves as a potential substrate for microbial fermentation into high-value bioproducts [15], the recalcitrant nature of its starch and lignocellulosic fractions within the peel matrix limits enzymatic accessibility and restricts overall conversion efficiency.

Recent studies have identified the black soldier fly larva (BSFL; *Hermetia illucens*) as a highly effective biological converter capable of transforming diverse organic wastes, including kitchen scraps, livestock manure, and food processing by-products, into high-value biomass [16,17]. The specialized digestive enzymes of BSFL rapidly degrade the starch-rich components of potato peel waste, synthesizing larval protein and fat while generating frass that contributes to the soil carbon cycle [18]. This insect-mediated process facilitates a low-carbon circular economy by significantly reducing waste mass and suppressing key pathogens, such as *Salmonella* spp. and *Escherichia coli* [19]. Consequently, the resulting nutrient-dense biomass serves as an environmentally sustainable alternative to conventional proteins in poultry, aquaculture, and livestock feed [20,21].

The work was driven by three objectives: (1) to determine the optimal compound ratio of potato peel waste for enhancing the conversion of antibiotic fermentation residue by BSFL, (2) to define the larval stocking density that maximizes waste reduction without compromising biomass quality, and (3) to verify the safety and nutritional value of the resulting larval meal for poultry, aquaculture, and livestock, thereby converting a hazardous waste stream into a marketable protein ingredient. Notably, according to Standard No. 246 issued by the Ministry of Agriculture and Rural Affairs of China, nosiheptide has been reclassified from a feed additive to a veterinary drug and can no longer be arbitrarily added to feed; it may only be used as a veterinary therapeutic agent.

## 2. Materials and Methods

### 2.1. Sources of Black Soldier Fly, Antibiotic Fermentation Residue, and Potato Peel Waste

The BSFL utilized in this study originated from the black soldier fly Wuhan strain [22]. These larvae were cultivated at the National Key Laboratory of Agricultural Microbiology, Huazhong Agricultural University, located in Wuhan, China. The substrate employed in this research, nosiheptide residue, was extracted from nosiheptide fermentation conducted by Gansu Huirui Fermentation Technology Research Institute Co., Ltd., Lanzhou, China, and potato peel waste was collected from potato processing plants. After hatching, the larvae were fed according to standard protocols for the first five days. Feeding was terminated on day 5, and the larvae were separated from the feed on day 6 for subsequent conversion experiments.

### 2.2. Analysis Physical and Chemical Composition

The carbon-to-nitrogen ratio (C:N) is a critical indicator for substrate nutrient balance. Total nitrogen (TN) and total organic carbon (TOC) in antibiotic fermentation residue and potato peel waste were quantified using an elemental analyzer. Dissolved organic carbon (DOC) was similarly measured following acidification to remove inorganic carbon.

For TN determination, 0.5 g of each sample was digested with K_2_SO_4_, CuSO_4_, and 10 mL of concentrated H_2_SO_4_ using a Hanon HS220F digestion system. The digestion program consisted of three stages: 160 °C for 30 min, 280 °C for 60 min, and 420 °C for 60 min. Upon cooling, the TN content was quantified using a Hanon K1160 automatic Kjeldahl nitrogen analyzer (Hanon Advanced Technology Group Co., Ltd., Jinan, China).

Determination of crude fat content: The dried insect bodies were ground into a fine powder. An accurately weighed 2 g portion of the powder was placed into a filter paper, which was then folded to prevent any leakage, and the total weight of the paper plus sample was recorded. The filter paper tube containing the sample was placed into the extraction cylinder of a Soxhlet apparatus. A lipid extraction flask, which had been previously dried and brought to constant mass, was attached to the unit. Petroleum ether with a boiling point range of 60 to 90 °C was introduced through the upper end of the condenser until the solvent volume reached approximately two thirds of the flask capacity. The condenser water flow was initiated, and the Soxhlet apparatus was heated using a water bath maintained at 80 °C to conduct extraction for a duration of 8 h. Following the completion of isothermal drying, it was weighed until a constant mass was achieved to allow for gravimetric calculation of the crude fat content.

Fat content (%) = (Total weight before extraction − Total weight after  extraction)/Weight of insect powder × 100%

### 2.3. Experimental Design for Identifying the Optimal Formulation for Co-Conversion of Antibiotic Fermentation Residue and Potato Peel Waste

To accommodate the significant differences in physical states between the antibiotic fermentation residue and potato peel waste, a series of experimental diets were formulated with varying proportions of these materials while maintaining a constant total dry weight (Table 1). Specifically, 200 g of the substrate mixtures were prepared according to the designated ratios and placed into small bucket bioreactors. Each bioreactor was inoculated with 200 6-day-old BSFL, and all treatments were performed in triplicate. Concurrently, the baseline amounts of available protein and carbohydrates allocated per larva under each corresponding dietary condition were calculated. The feeding trials were conducted inside a greenhouse environment maintained at 28 ± 2 °C and with a relative humidity of 60% to 70%. The experimental period was initiated immediately upon the emergence of the first prepupae within the rearing containers. Subsequently, the larvae were manually separated from the substrate, thoroughly washed with distilled water, and sacrificed by thermal inactivation in hot water at 100 °C. The larvae were then dried at 65 °C for a duration of two days, after which various conversion metrics were calculated based on the weight of the lost substrate and the weight gain of the larvae.

### 2.4. Effects of Different Ratio of Larval Number to Feed on the Conversion of Antibiotic Fermentation Residues from BSFL and the Degradation of Nosiheptide

Previous experiments indicated that the initially established ratio of larval numbers to feed did not fully optimize substrate utilization. Concurrently, preliminary small-scale experiments were conducted to modify the larva-to-feed ratio, which significantly enhanced the conversion efficiency of BSFL with respect to antibiotic fermentation residue. Consequently, optimal conditions were determined by establishing various gradients of larva-to-feed ratios, aimed at improving the overall conversion efficiency of the substrate and facilitating the degradation of nosiheptide in antibiotic fermentation residue, as shown in Table 2.

### 2.5. Evaluation of the Safety and Nutritional Value of the BSFL

Safety evaluation: Two treatment groups were established with wet weight ratios of antibiotic fermentation residue to potato peel waste of 1:6 (high) and 1:15 (low). The 1:15 group represented the optimal ratio of antibiotic fermentation residue to potato peel waste during the early stage of BSFL conversion, whereas the 1:6 group contained the maximum concentration of antibiotic fermentation residue that did not significantly impair BSFL conversion performance. The establishment of these two groups facilitated the safety assessment of the resulting BSFL biomass following the bioconversion of antibiotic fermentation residue waste. This assessment primarily involved: (1) determination of nosiheptide concentration in the BSFL bodies after transformation. Upon completion of the feeding trials, 30 BSFL were collected from each group. The larval external surfaces were rinsed with distilled water, and the larvae were then placed in 200 mL of clean water and allowed to stand for 24 h, with water being renewed every 12 h. Larvae were sampled at 24 and 48 h, respectively, and the nosiheptide content in their bodies was measured, and (2) consideration of the presence of nosiheptide in the crude protein of BSFL after the processes of drying, grinding, and degreasing, given the potential use of BSFL as a protein source in animal feed.

Resource utilization assessment: The fatty acid and amino acid composition and content of the two groups of BSFL, raised with differing concentrations of antibiotic fermentation residue, were determined and compared with those of BSFL reared on alternative organic waste as a reference. This analysis aimed to elucidate the nutritional composition of BSFL cultivated with antibiotic fermentation residue and to evaluate the feasibility of its practical applications, particularly in relation to feed utilization The determination of amino acids [23] and fatty acids [24] in the samples was carried out in accordance with the national standards of the People’s Republic of China.

### 2.6. DNA Extraction and Sequencing

The total microbial DNA from the samples was extracted utilizing the phenol chloroform method. The concentrations of the extracted DNA were quantified using a Nanodrop 2000 spectro-photometer (Thermo Scientific, Wilmington, DE, USA). The DNA samples were subsequently fragmented by sonication to an average size of 350 bp. Following fragmentation, the DNA fragments underwent end polishing, A tailing, and ligation with full-length adapters for Illumina sequencing, accompanied by further PCR amplification. Ultimately, the PCR products were purified using the AMPure XP system (Beckman Coulter, Brea, CA, USA), and the resulting libraries were evaluated for size distribution employing an Agilent 2100 Bioanalyzer (Agilent Technologies, Santa Clara, CA, USA) and quantified through real-time PCR.

The total microbiome of the BSFL intestines on day 0 was designated as group G0. On the 8th day following bioconversion with high and low concentrations of nosiheptide, the intestinal microbiomes of the BSFL were designated as HG8 and LG8, respectively. The terms HM0 and LM0 represented the total microbiomes within the substrate matrices of the high- and low-concentration nosiheptide experimental groups at the initiation of the bioconversion process. Similarly, HM8 and LM8 were the total microbiomes in the substrate matrix of the high- and low-concentration nosiheptide experimental groups after 8 days of transformation. The sequence information after sequencing was analyzed using the bioinformatics analysis platform of Shanghai Meiji Biotechnology, Shanghai, China.

### 2.7. Extraction and Detection of Nosiheptide in a Mixed Material Matrix

Following dehydration, the preserved samples were ground using a grinding machine and sieved through a 40-mesh sieve. An amount of 0.2 g of the crushed sample was transferred to a 15 mL centrifuge tube, to which 2 mL of phosphate-buffered solution and 8 mL of dimethylformamide (DMF) were added. The mixture was vortexed at 2500 rpm for 30 s and subsequently subjected to ultrasonic extraction for 20 min. During the sonication process, the mixture was vortexed for 15 s at 5 min intervals. After extraction, the sample was centrifuged at 8000 rpm for 10 min. The supernatant was collected and filtered through a 0.22 µm filter membrane into a 2 mL injection vial for liquid chromatography analysis.

The liquid chromatography conditions were as follows: mobile phase A was 0.025% phosphoric acid aqueous solution, and mobile phase B was acetonitrile (chromatographically pure). Elution was performed with a gradient of 50% B. The detection wavelength was set to 241 nm. The mobile phase flow rate was 1 mL/min, and the injection volume was 20 µL. The column temperature was maintained at 30 °C. The separation column was a Venusil XBP C18 column (4.6 × 250 mm, 5 μm) from Agela Technologies, Tianjin, China.

Preparation and spiking procedure: Nosiheptide (5 mg) was dissolved in 5 mL of acetonitrile to obtain a 1 mg/mL stock solution, which was then serially diluted with acetonitrile to a 0.2 mg/mL standard solution. The dried insect powder was ground, and 0.2 g of the powder was accurately weighed and transferred into a 15 mL centrifuge tube. Then, 200 μL of the 1 mg/mL and 0.2 mg/mL standard solutions were added, respectively. The mixture was incubated at room temperature for 12 h. The nosiheptide content in the samples was determined according to the extraction and detection method described above.

Recovery rate = (spike value − background value)/actual added amount × 100%

### 2.8. Antibacterial Test of Antibiotic Residues Before and After Transformation

Take 0.5 g of antibiotic residues from the H and L groups before and after conversion and place them in a 2 mL centrifuge tube. Add 1 mL of sterile water, vortex for 30 s, and then filter. Then filter again using a sterile 0.22 μm filter tip. Place an Oxford cup on a plate containing Bacillus spores and add 100 μL of the filtered antibiotic solution into the Oxford cup. Incubate the plate at 37 °C for 12 h. Measure the diameter of the inhibition zone using a caliper.

### 2.9. Calculation and Statistical Analysis

The material reduction rate, conversion rate, and feed conversion ratio are determined utilizing the following formulas:

Material reduction rate = (W1−W2)/W1 × 100% (1)

W1 is the initial weight of the material, W2 is the weight of the residue after conversion.

Bioconversion rate = dry weight increment of larvae/total amount of added materials × 100%(2)

Feed conversion rate = total feeding amount/larval increment(3)

The crude protein content = the total nitrogen content × 6.25(4)

Statistical analysis was conducted using SPSS 27.0 software. The experimental results were analyzed using a one-way analysis of variance (ANOVA) to compare the significance (P) of the differences between the means of each group. The difference is considered statistically significant with *p* < 0.05. All experiments were conducted in triplicate.

## 3. Results

### 3.1. The Formula Optimization for Antibiotic Fermentation Residue and Potato Peel Waste Mixed Conversion by BSFL

On day 12 of the experiment, the first prepupae were observed in the R-5 group, indicating that the experimental period was nearing completion. Across all treatments, larval biomass decreased in a near-linear fashion as the proportion of antibiotic fermentation residue increased (Table 3). The optimal formulation, R-8, which contained the highest proportion of potato peel waste, yielded 6.82 ± 0.10 g of dry larval mass and consumed 22.41 ± 0.29 g of substrate. In contrast, formulations with higher antibiotic fermentation residue content exhibited significantly lower performance. The reduction l is attributed to the compact texture, poor palatability, and residual antibiotic load of the residue, which collectively inhibited BSFL feeding and reduced overall bioconversion efficiency.

**Figure 1:**
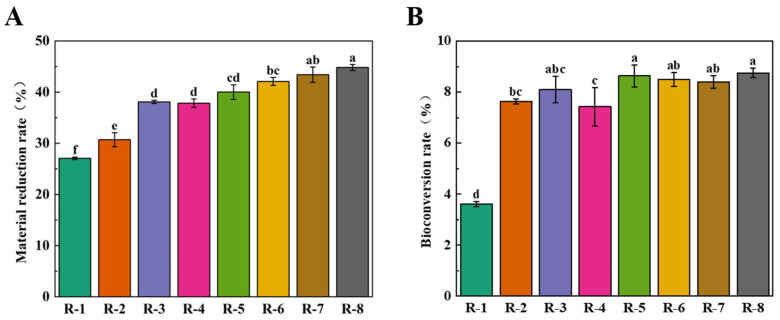
Material reduction rate (**A**) and bioconversion rate (**B**) of the mixture of BSFL converted antibiotic fermentation residue and potato peel waste. Different lower-case letters indicate significant difference (*p* < 0.05).

An integrated assessment of material reduction and bioconversion efficiency for BSFL reared on antibiotic fermentation residue and potato peel waste demonstrated that both the R-5 and R-8 treatments outperformed all other blends. From an operational perspective, R-8 represents the more favorable option; its bioconversion rate was comparable to that of R-5, yet it achieved a higher substrate mass loss (Figure 1A,B). Practical application indicates that for a given initial feed weight, R-8 larvae consume more substrate and generate a greater dry larval yield.

### 3.2. Degradation Efficiency of Nosiheptide by BSFL

The results of the nosiheptide spiking experiment demonstrated a high recovery rate for the extraction method, thereby confirming its reliability (Table 4).

**Figure 2:**
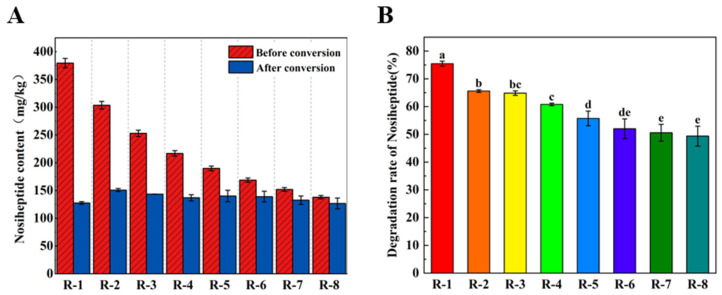
The degradation ability of BSFL towards nosiheptide. (**A**) The concentration of nosiheptide in the residues before and after BSFL conversion process. (**B**) The degradation rate of nosiheptide by BSFL is presented. Error bars represent standard deviations calculated from triplicate samples. Different lower-case letters indicate statistically significant differences (*p* < 0.05).

Following bioconversion, residual nosiheptide was quantified using high-performance liquid chromatography. Groups R-1 to R-8 were formulated with a descending gradient of antibiotic fermentation residue at an equal total dry mass, resulting in a corresponding decrease in the initial antibiotic load. After conversion, the nosiheptide content in the residue of each treatment group decreased to similar levels, exhibiting no statistically significant differences among the groups. The lowest concentration was 126.68 ± 9.77 mg/kg in the R-8 group, whereas the highest concentration was 150.76 ± 2.79 mg/kg in the R-2 group (Figure 2A).

Analysis of the nosiheptide degradation rates revealed a positive correlation with the proportion of antibiotic fermentation residue. The highest degradation rate (75.47 ± 0.87%) was observed in the R-1 group, whereas the lowest rate (49.38 ± 3.59%) was found in the R-8 group (Figure 2B). This difference may be attributed to insufficient nutrient availability in the groups containing a higher proportion of antibiotic fermentation residue. Because the palatability of the antibiotic fermentation residue is poor for BSFL, it is likely consumed minimally to sustain basic survival, resulting in low bioconversion efficacy. Conversely, as the proportion of antibiotic fermentation residue decreases, the availability of more nutrients and easily digestible co-substrates increases. This shift may lead to a reduced relative consumption of the antibiotic fermentation residue, thereby contributing to the lower degradation rates of nosiheptide. Furthermore, due to the lower moisture content of the residue, the larvae could be separated with greater ease.

Considering the comprehensive bioconversion rate and the degradation rate of nosiheptide, the conversion performance parameters of R-5 outperformed those of the other treatment groups.

### 3.3. The Effect of Larvae-to-Feed Ratio on the Growth Performance of Black Soldier Fly Larvae and Nosiheptide Degradation Capability

**Figure 3:**
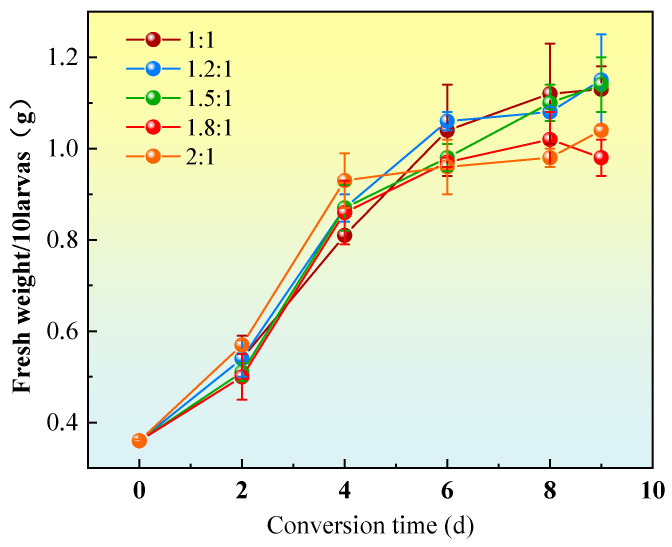
Influence on growth of BSFL converted antibiotic fermentation residue and potato peel waste under different ratio of larva number to feed. Error bars are the standard variations for triplicate samples.

By day 8 of bioconversion, larval fresh weight peaked across all treatment groups. During the initial four days, growth rates were similar across groups. However, between days 4 and 8, groups 4 (1.8:1 larva-to-feed ratio) and 5 (2:1) exhibited slower growth than groups 1 to 3. Consequently, on day 9, the fresh biomass of BSFL in groups 4 and 5 was significantly lower than that observed in the other groups (Figure 3).

**Figure 4:**
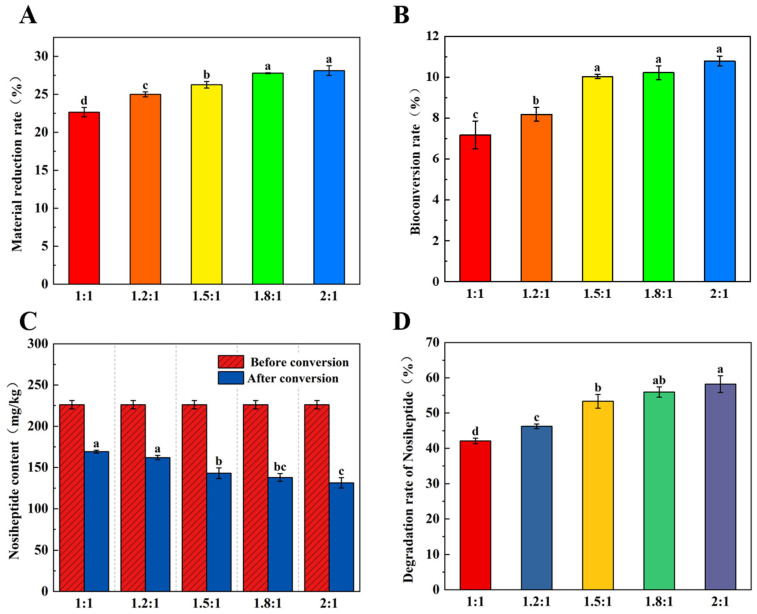
The effect of larvae-to-feed ratio on the growth performance of black soldier fly larvae and nosiheptide degradation capability. (**A**) The material reduction rate after BSFL conversion. (**B**) The bioconversion rate of BSFL after conversion. (**C**) The content of nosiheptide in the residues before and after BSFL conversion. (**D**) The degradation rate of nosiheptide by BSFL. Different lower-case letters indicate significant difference (*p* < 0.05).

Increasing the larva-to-feed ratio from 1:1 to 2:1 improved the bioconversion performance of the antibiotic residue and potato peel blend, with the maximum material reduction (28.13 ± 0.65%) and bioconversion rate (10.79 ± 0.24%) observed at the 2:1 ratio. Further increasing the ratio to 1.8:1 and 2:1 led to only a modest increase in substrate consumption and antibiotic degradation (Figure 4), whereas the higher stocking density yielded lighter, more variable larvae (Figure 3). Balancing biomass quality, process efficiency and economic viability, the 1.5:1 ratio emerges as the optimal condition, achieving a material reduction of 26.27% and a bioconversion rate of 10.03%.

Statistical analysis of residual nosiheptide demonstrated that larval activity exerts a significant impact on antibiotic degradation. At a 2:1 larva-to-feed ratio, degradation peaked at 58.21 ± 2.38%, which was significantly higher than that in any other treatment (Figure 4D), coinciding with the greatest substrate loss and bioconversion efficiency (Figure 4B). This ratio therefore represents the upper limit for nosiheptide removal. However, the same high density regimen reduced larval fresh weight well below that achieved at lower stocking densities, indicating a trade-off between maximal antibiotic degradation and optimal biomass yield (Figure 3). Increasing the larva-to-feed ratio from 1.2:1 to 1.5:1 produced a clear, statistically significant increase in both bioconversion and nosiheptide degradation (Figure 4B,D). When the ratio was further increased to 1.8:1 and 2:1, the rate of increase for both substrate consumption and antibiotic degradation decelerated. This plateau occurs once the fixed substrate can no longer meet the collective nutritional demand; beyond this “nutrient ceiling” the larvae cannot sustain proportional improvements in conversion or antibiotic removal. Consequently, the 1.5:1 ratio provides the optimal balance between performance and economic return.

### 3.4. Safety Assessment of Feeding BSFL with Antibiotic Fermentation Residue

Antibacterial assays demonstrated that BSFL transformation significantly reduced the inhibitory effects of fermentation residues, suggesting the conversion of nosiheptide into lower-toxicity metabolites (Table 5). BSFL performance varied across dilution ratios (Table 6); larvae reared on a 1:15 substrate (low concentration) exhibited superior fresh-weight gain, achieving a material reduction of 35.82% and a bioconversion rate of 15.16%. Compared with larvae fed the high-load substrate, the low-load group boosted material reduction and bioconversion by 65.99% and 61.96%, respectively.

**Figure 5:**
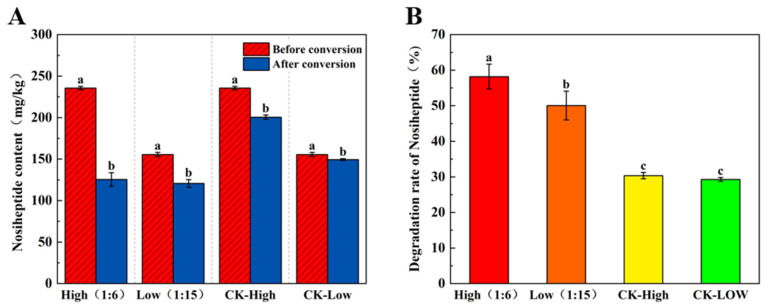
The degradation ability of BSFL towards nosiheptide. (**A**) The concentration of nosiheptide in the residues before and after the conversion process by BSFL. (**B**) The degradation rate of nosiheptide facilitated by BSFL. The CK group represents the high- or low-concentration antibiotic residues group without black soldier fly. Error bars represent the standard deviation of triplicate samples. Different lower-case letters indicate significant differences (*p* < 0.05).

HPLC analysis of pre- and post-treatment samples demonstrated that the high concentration mixture (1:6) achieved faster antibiotic clearance, degrading 58.18 ± 3.51% of nosiheptide compared with 50.05 ± 4.04% in the 1:15 dilution. This significant difference underscores the capacity of the larvae to tolerate and process highly concentrated residue. In the natural composting controls, CK-High and CK-Low, which were loaded with high and low concentrations of nosiheptide respectively, but devoid of larvae, antibiotic losses reached only 30.34 ± 0.89% and 29.28 ± 0.54%. These results confirm that BSFL significantly accelerate degradation compared to passive composting (Figure 5).

**Figure 6:**
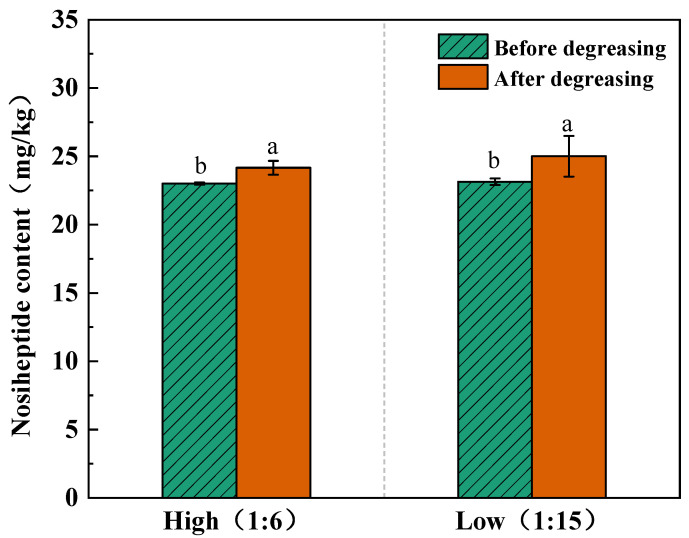
After 24 h of intestinal emptying, the nosiheptide content of BSFL was assessed both after and before degreasing process. Different lower-case letters indicate significant differences (*p* < 0.05).

Residual antibiotic levels in the final insect product were minimal and stable. Following gut clearance, the larval protein meal contained 23.00 ± 0.08 mg/kg (1:6 substrate) and 23.14 ± 0.24 mg/kg (1:15 substrate), which increased only marginally to 24.16 ± 0.51 and 25.01 ± 1.49 mg/kg after solvent defatting (Figure 6). Although degreasing caused a slight upward shift in nosiheptide concentration due to mass loss from lipid extraction, this increment was statistically non-significant, confirming that fat removal does not meaningfully elevate antibiotic concentration in the finished larval powder. Notably, after 48 h of fasting, the nosiheptide residues were below the detection limit in the bodies of the BSFL, indicating that an adequate gut emptying period successfully reduces antibiotic accumulation to negligible levels.

### 3.5. Changes in Microbial Communities

#### 3.5.1. Bacterial Richness and Diversity

**Figure 7:**
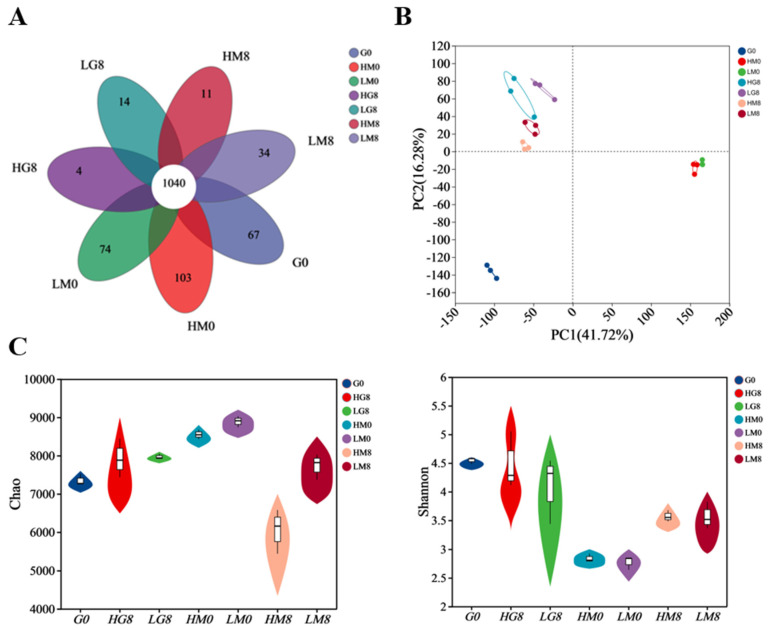
(**A**) Venn diagram analysis, the numbers indicate the corresponding number of species; (**B**) Principal component analysis based on the Bray–Curtis distances; (**C**) Chao1 richness and Shannon index.

Venn analysis revealed significant shifts in microbial OTU distribution across treatments. Raw substrates (HM0, LM0) and initial larval gut samples (G0) exhibited the highest OTU richness; however, nosiheptide residue acted as a potent selective pressure, markedly reducing richness in post-conversion residues (HM8, LM8) and gut samples (HG8, LG8). This reduction followed a dose-dependent trend, with high-concentration treatments (HM8, HG8) maintaining substantially lower out counts than low-concentration treatments (LM8, LG8). Despite these shifts and the antibiotic gradient, a core microbiome of 1040 OTUs persisted across all samples, indicating a resilient microbial community (Figure 7A).

Chao1 and Shannon index analyses revealed that following BSFL processing of the nosiheptide residue, gut microbial richness remained statistically stable, whereas diversity decreased slightly. This pattern reflects the selective pressure exerted by the antibiotic; residual nosiheptide promotes the enrichment of resistant taxa, thereby reducing species evenness without substantially affecting total species richness. Functionally redundant populations, which became dominated by antibiotic-tolerant lineages, continue to decompose organic matter. Consequently, richness was maintained despite these shifts in community structure (Figure 7C).

Principal Component Analysis (PCA) revealed distinct clustering patterns, with PC1 and PC2 accounting for 41.72% and 16.28% of the total variance, respectively. Although BSFL gut microbiota and substrate samples initially formed discrete clusters with divergent compositions, they exhibited marked convergence following the bioconversion process (Figure 7B). These findings suggest that the feeding substrate significantly influences the microbial composition in the BSFL gut.

#### 3.5.2. Composition of Bacterial Community

**Figure 8:**
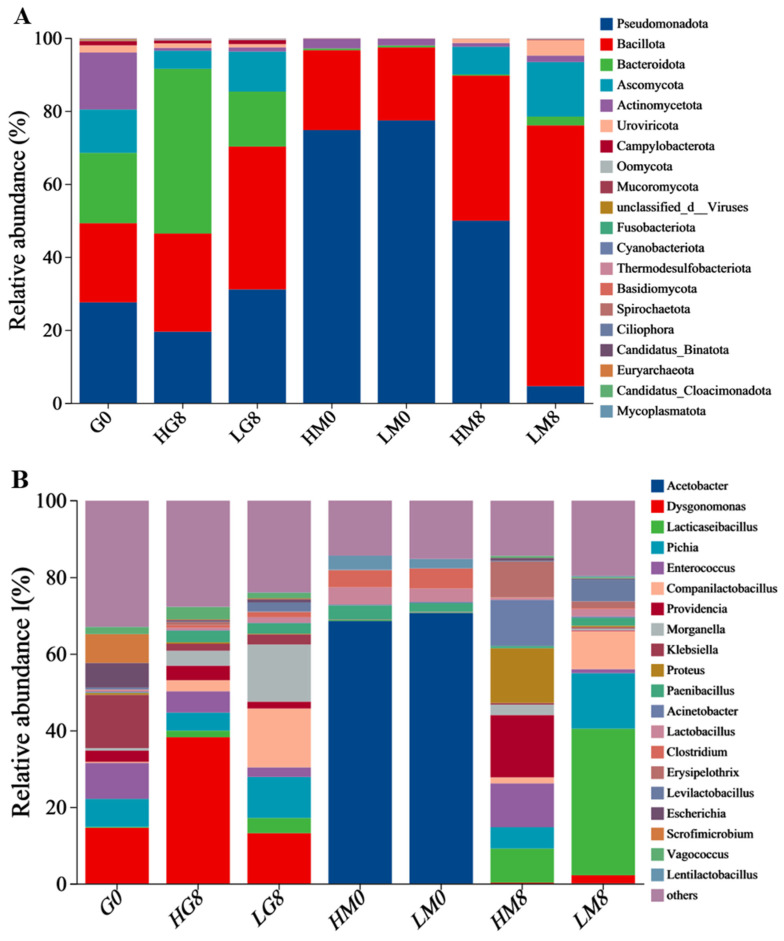
Microbial community composition in the gut of BSFL and associated substrate. (**A**) Phylum levels; (**B**) Genus Levels.

During the bioconversion of antibiotic-laden residues, the BSFL gut microbiome underwent concentration-dependent successions at the phylum and genus levels, driven by the interplay of substrate composition and antibiotic stress. In untreated larvae (G0), the community was co-dominated by Pseudomonadota (27.56%), Bacillota (21.70%) and Bacteroidota (19.27%) co-dominated. High-concentration treatments (HG8) induced a sharp shift toward Bacteroidota (45.13%), specifically the genus *Dysgonomonas* (38.20%), while the corresponding substrate (HM8) became dominated by Pseudomonadota (49.94%) and enriched with *Enterococcus*. In contrast, low-concentration conditions fostered distinct taxonomic profiles: the low-concentration gut group (LG8) showed an increased Bacillota abundance (39.15%), accompanied by the proliferation of yeast (Pichia, 10.70%) and probiotic species (Companilactobacillus, 15.36%), while the LM8 substrate was dominated by Bacillota (71.43%), primarily Lacticaseibacillus (38.26%). These results indicate that high antibiotic pressure selects for resistant, specialized lineages, whereas lower concentrations promote a probiotic-rich consortium of lactic acid bacteria and yeast (Figure 8A).

Genus-level analysis revealed that *Dysgonomonas* (Bacteroidota) became the predominant taxon (38.20%) in the high-concentration gut group (HG8), representing a 2.9-fold increase over the low-concentration group (LG8) and highlighting its robust antibiotic tolerance. Conversely, the LG8 group maintained higher functional diversity, characterized by the co-occurrence of *Dysgonomonas* (13.16%), the probiotic *Companilactobacillus* (15.36%), the fermentative yeast *Pichia* (10.70%), and nitrogen-cycling *Morganella* (14.91%). Microbial succession was more pronounced in the substrates; while initial samples (HM0/LM0) were dominated by *Acetobacter* (68.58–70.66%), post-conversion residues exhibited a sharp divergence. The HM8 group accumulated a resistant consortium, primarily *Providencia* (16.27%), *Proteus* (14.32%), totaling 63.38% abundance. In contrast, the LM8 group developed a synergistic fermentation symbiosis dominated by *Lacticaseibacillus* (38.26%) and *Pichia* (14.50%), indicating that lower antibiotic concentrations favor the establishment of beneficial probiotic communities (Figure 8B).

### 3.6. Assessment of Resource Utilization of Antibiotic Fermentation Residue Feeding BSFL

Crude protein content serves as an estimate of the total protein levels in feed, whereas the amino acid profile indicates protein quality [25], particularly regarding the balance between essential and nonessential amino acids. Statistical analysis of crude protein, crude fat, and amino acids in BSFL reared on highly concentrated and low-concentration antibiotic fermentation residue showed that variation in the proportions of antibiotic fermentation residue and potato peel waste resulted in significant differences in crude protein and crude fat contents between the high (1:6) and low (1:15) groups. The high group exhibited higher crude protein and lower crude fat contents (35.64 ± 0.66% and 25.03 ± 0.60%, respectively) (Table 7). Therefore, formulation ratios should be selected based on specific production requirements. BSFL reared on antibiotic fermentation residue are nutritionally rich and exhibit a uniform amino acid distribution that closely matches the amino acid profiles and major amino acid levels of larvae fed on kitchen waste, as reported in the literature [26]. Adequate concentrations of glycine and proline are recognized as indispensable for maximizing animal health and growth [27]. In larvae reared on the high-load (1:6) antibiotic residue diet, these two amino acids each surpass 1.4%, whereas most other amino acids were lower than those in the low-load (1:15) group; only methionine, histidine, phenylalanine and lysine occurred in higher concentrations under the high load regime. Regardless of the antibiotic load level, glutamic acid, aspartic acid and alanine dominated the amino acid profile. Specifically, glutamic acid exceeded 3% prior to degreasing, which may enhance feed aroma and palatability. Meanwhile, key essential amino acids, including lysine, threonine, valine and methionine, were maintained at standard feed specification levels.

The fatty acid profiles of BSFL reared on both loading levels were characterized by lauric, palmitic and oleic acids as the primary components, with their proportions influenced by substrate composition. Larvae from the high-load (1:6) diet delivered a total fatty acid pool dominated by lauric acid (36.76% versus 24.83% in the 1:15 group), while oleic acid levels were significantly reduced. Linoleic and myristic acids followed, representing 7.54% and 6.82% in the 1:6 group compared to 11.16% and 5.35% in the 1:15 group (Table 7). Lauric acid, the dominant fatty acid in BSFL, has known antibacterial, antiviral and anticancer activity, making it valuable in the pharmaceutical, food and cosmetics industries. Its concentration in BSFL is comparable to that in coconut and palm kernel oils, positioning BSFL as a sustainable alternative feedstock for industries currently dependent on traditional lauric crops [28]. Moreover, the total unsaturated fatty acid fraction, which comprised oleic (18:1), linoleic (18:2), palmitoleic (16:1), linolenic (18:3) and arachidonic (20:1) acids, accounted for 36.79% in the low-load (1:15) group and 48.79% in the high-load (1:6) group (Table 8).

## 4. Discussion

Our findings demonstrate that the utilization of black soldier fly larvae provides an effective, integrated approach for both the valorization of antibiotic fermentation residues and the degradation of residual nosiheptide.

Traditionally, antibiotic fermentation residues have been disposed of via composting or landfilling, which represent low-value options that release antibiotics and greenhouse gases into the environment. Although most insect-based studies have focused on kitchen scraps, animal manures, and agro-industrial byproducts [29], recent work has shown that BSFL can also degrade a range of veterinary antibiotics, including tetracycline [10], ciprofloxacin [30], and oxytetracycline [11]. The present study, therefore, investigated the potential of BSFL to process antibiotic fermentation residues, aiming to enhance their value and reduce antibiotic residues. Preliminary experiments revealed that BSFL could not directly utilize nosiheptide fermentation residue as the sole substrate, a finding not previously documented. It is hypothesized that the concentration of secondary metabolites, particularly nosihetide and other inhibitory substances present in the residue, exceeds the tolerance threshold of BSFL. To address this limitation, we proposed the incorporation of an additional organic waste source, specifically potato peel waste, which is a byproduct of potato processing. This co-digestion approach serves two purposes: first, it dilutes the proportion of nosihetide fermentation residue, thereby improving palatability for the larvae; second, it supplements the overall nutritional profile of the material, promoting BSFL conversion efficiency.

To optimize conversion efficiency, low-cost potato peel waste was incorporated to facilitate the effective treatment of the antibiotic fermentation residue [31]. The evaluation began with the highest proportion of antibiotic fermentation residue that the larvae could tolerate, followed by a stepwise reduction in the ratio to identify the most effective blend with potato peel waste. As shown in Table 3 and Figure 4, this optimal mixture maximized BSFL performance, delivering the greatest larval biomass, substrate reduction, and overall bioconversion rate. Co-digestion enhances yields due to an improved nutrient balance and buffering capacity, which are essential for establishing positive growth synergies [32]. Across all blends, BSFL successfully removed the residual nutrients from the *Streptomyces* fermentation residue and concurrently reduced residual nosiheptide to acceptable levels. It is worth noting that when determining the crude protein content in waste substrates, the total nitrogen content measured via the Kjeldahl method may encompass nonprotein nitrogenous substances that are difficult for the larvae to utilize, such as urea, thereby leading to an overestimation of the actual nutritional protein content. Consequently, a combination of the trichloroacetic acid (TCA) precipitation method and the Kjeldahl method will be employed in subsequent analyses to ensure accurate true protein quantification.

To maximize the valorization of antibiotic residue and potato peel waste while accelerating nosiheptide removal, a bench-scale BSFL process was developed focusing on stocking density as the critical variable. Optimization of the larva-to-feed ratio improved substrate utilization, material reduction, and antibiotic degradation (Figure 3 and Figure 4). While increasing the ratio from 1:1 to 2:1 enhanced overall conversion, diminishing returns were observed beyond 1.5:1; further increases in density failed to improve dry weight gain, bioconversion rate, or feed conversion efficiency, instead leading to reduced individual larval weight. Based on throughput and production costs, a ratio of 1.5:1 was identified as the optimal stocking density (Figure 3). Discrepancies in BSFL performance at a 1:1 ratio between experimental phases likely resulted from batch variations in potato peel quality and fluctuations in ambient conditions. As feed consumption and weight gain typically peak at 30 °C and 65 to 75% relative humidity [33], minor deviations from these environmental optima may explain the observed inconsistencies in conversion efficiency.

With the pilot-scale conversion of antibiotic fermentation residue by BSFL optimized, the subsequent step is to verify the safety and nutritional quality of the resulting larvae for application in livestock and aquaculture feed. Therefore, high and low residue treatments were evaluated, quantifying residual nosiheptide in both the harvested larvae and subsequent defatted crude protein meal to ensure compliance with feed safety standards. Furthermore, the comprehensive nutritional profiles, including crude protein, fat, amino acid and fatty acid spectra, were compared between larvae reared on high (1:6) and low (1:15) antibiotic residue blends to assess the suitability of the resulting meal as a sustainable feed protein source. Previous work has shown that following the bioconversion of oxytetracycline fermentation residue, no residual oxytetracycline or tissue accumulation was detected in BSFL [11]. The low but measurable nosiheptide levels reported in the present study likely reflect incomplete gut emptying rather than true tissue bioaccumulation; after a longer period of intestinal emptying, no residues of nosiheptide were detected in the larval body.

Microbial community restructuring suggests that antibiotic concentration is a primary driver of BSFL conversion ecosystems. High-concentration residues exert dual selective pressures: antibiotic stress eliminates sensitive taxa such as *Lactobacillus*, while high-nitrogen, and low-carbon levels favor nitrogen-tolerant, resistant lineages. Conversely, low-concentration treatments, buffered by starch-rich potato peel waste, shift the metabolic balance toward acidogenic fermentation, allowing probiotics to dominate the niche. This divergence highlights the synergistic interaction between substrate characteristics (C/N ratio and antibiotic concentration) and microbial adaptability. These findings provide a theoretical framework for optimizing waste formulations and targeted bioaugmentation using *Lactobacillus* and yeast consortia to enhance bioconversion efficiency and safety.

This study assesses the nutritional value of BSFL reared on antibiotic fermentation residue. The amino acid profile of BSFL meal is similar to that of fishmeal, rendering it a highly suitable protein source for animal feed relative to other insect-based alternatives [25]. Additionally, the high protein content and balanced amino acid composition contribute to enhanced feed utilization efficiency and overall feed quality [34]. In the present study, valine, lysine, leucine and arginine dominated the essential amino acid signature of BSFL reared on the antibiotic residue and potato peel blend, echoing previous reports that lysine, valine and arginine are consistently the most abundant essentials amino acids regardless of waste substrate [35]. Although the total essential amino acid complement is modest relative to conventional fish or soybean meal, several individual amino acids match or exceed those benchmarks. Glutamic acid, for example, surpasses 3%, which is higher than the 2.75% recorded for chicken manure-fed larvae [36], and acts as a natural flavor enhancer that can improve feed palatability. The fatty acid profiles obtained are consistent with literature values for BSFL reared on other organic wastes [26]. Lauric acid, palmitic acid, and oleic acid were identified as the primary fatty acid components in BSFL [35,37,38]. Research indicates that lauric acid in BSFL provides protection against lipid oxidation and enhances larval tolerance to elevated temperatures [39]. In this study, the lauric acid content in the High (1:6) group represented a substantial proportion of total fatty acids, falling within the typical range reported for BSFL meal derived from various organic wastes. Lauric acid is recognized for its antibacterial and antiviral properties and is widely utilized in the pharmaceutical, food, and chemical industries [28]. Attention should also be directed to the unsaturated fatty acids, which comprised a considerable portion of the total fatty acid pool, exhibiting notably high levels of oleic acid (18:1), linoleic acid (18:2), and palmitic acid (16:1) compared to previous reports [40].

Compared with incineration, bioconversion using BSFL converts organic carbon in waste into storage forms such as proteins and fats, whereas incineration releases most of the total carbon into the atmosphere as carbon dioxide. Therefore, bioconversion results in partial carbon fixation. Regarding the degradation and residue treatment of nosiheptide in practical applications, publicly available industrial-scale research data on its degradation remain limited. Based on existing literature, current treatment strategies focus on two primary aspects: process optimization to minimize waste emissions at the production source, and the reliable analysis, detection, and safe disposal of unavoidable antibiotic-laden waste. For instance, a Chinese patent describes a method in which fermentation wastewater from nosiheptide production is treated using a combination of biological processes, including a membrane bioreactor (MBR), to achieve water recycling. This approach reduces pollutant emissions at the source and represents a cleaner production technology. However, for nosiheptide that has already entered the environment, mature and specific industrial degradation schemes remain sparse in the public domain. In practice, common treatment strategies used for other antibiotics are often applied, such as membrane filtration, adsorption, and biodegradation [41]. The present study provides a critical framework for the eco-friendly management of nosiheptide fermentation residue. Through the synergistic action of microorganisms and insects, the residual nosiheptide in the fermentation residue was partially degraded, thereby reducing its potential release into the environment and subsequent pollution.

However, the structural characterization of nosiheptide degradation products was not conducted, which limits a comprehensive understanding of the metabolic pathways and molecular transformations involved. This limitation prevents a full understanding of the degradation pathways and the molecular transformations occurring during the treatment process. Determining the structural fate of nosiheptide is important for evaluating the safety of the treated residues. Specifically, confirming the complete loss of antimicrobial activity (i.e., detoxification) of the resulting products is necessary before any practical application. Without this confirmation, the possible persistence of bioactive intermediates or unknown transformation products cannot be excluded. Therefore, future research should systematically identify these degradation products using advanced analytical techniques such as high-resolution mass spectrometry (HRMS) and nuclear magnetic resonance (NMR) spectroscopy. These efforts are needed to support the use of the proposed treatment strategy for the safe management of antibiotic fermentation residues in real-world settings.

Meanwhile, the use of biodegradation processes may facilitate the dissemination antibiotic resistance genes (ARGs), a risk previously documented during the biological treatment of various antibiotics [42]. In this study, the BSFL intestinal tract harbored microbiota with notable tolerance to nosiheptide. Specifically, high concentrations of nosiheptide induced a proliferation of Bacteroidota within the larval gut and a high proportion of Pseudomonadota within the resulting frass. These shifts indicate a potential risk of ARG transmission. Future research should focus on the potential for horizontal transfer of resistance genes within the “waste–larvae frass” chain, as well as on strategies to inhibit the overgrowth of resistant bacteria in the larval gut by adjusting feed formulations, such as adding probiotics or plant extracts. In practice, combining biological and physical treatment methods may be used. For example, high-temperature treatment can inactivate drug-resistant microorganisms in both the larval gut and the matrix, thereby reducing the spread of resistance genes.

In conclusion, BSFL offer a distinct technical advantage for the simultaneous valorization of antibiotic fermentation residues and potato peel waste. Future work should isolate and deploy nosiheptide-degrading functional bacteria to accelerate larval breakdown of the antibiotic, thereby lifting overall process efficiency. Additionally, although this study provides a first estimate of residual nosiheptide levels in processed larvae, comprehensive safety assays are still required before meals derived from antibiotic residue-fed larvae can be confidently commercialized as feed additives.

## 5. Conclusions

The addition of potato peel waste enabled BSFL to better utilize hazardous nosiheptide fermentation residue and effectively degrade the residual antibiotic. In this study, under a dry-weight ratio of 3:5 (antibiotic residue to potato peel), BSFL achieved a relatively high degradation efficiency and bioconversion rate for nosiheptide. Specifically, the material reduction and bioconversion rates reached 40.02% and 8.63%, respectively, while the nosiheptide degradation rate was 55.74%. By optimizing the larva-to-feed ratio, the conversion capacity of BSFL for antibiotic fermentation residue and the degradation efficiency of nosiheptide were further increased to 58.21%. Highly concentrated antibiotic treatment induced significant microbial shifts within the larval gut; the abundance of Bacteroidota in the high-concentration gut group (HG8) increased to 45.13%, with *Dysgonomonas* emerging as the dominant genus (38.20%). This abundance was 2.9-fold higher than that in the low-concentration group (LG8). Nutritional analysis of BSFL reared on the antibiotic fermentation residue demonstrated that crude protein and crude fat contents ranged from 30.02% to 35.64% and from 25.03% to 32.65%, respectively, depending on the proportion of antibiotic fermentation residue incorporated into the diet. The BSFL contained seven essential amino acids, with glutamic acid exceeding 3%, which imparts an umami flavor similar to natural monosodium glutamate, thereby enhancing the taste and palatability of animal feed. Furthermore, the unsaturated fatty acid content ranged from 36.79% to 48.79%. Collectively, these findings present a novel and effective strategy for the eco-friendly management and valorization of antibiotic fermentation residue waste utilizing BSFL.

## Figures and Tables

**Table 1:** Different compounding ratios of antibiotic fermentation residue and potato peel waste.

Group	Dry Weight Ratio (AFR/PPW)	Wet Weight Ratio (AFR/PPW)	C/N	Carbohydrate/Larva (mg/Larva)	Protein/Larva (mg/Larva)
R-1	3:1	1:3	13.70	134.30	52.29
R-2	3:2	1:6	13.47	141.56	54.90
R-3	1:1	1:9	13.34	146.39	56.63
R-4	3:4	1:12	13.30	149.84	57.87
R-5	3:5	1:15	13.18	152.43	58.80
R-6	1:2	1:18	13.13	154.45	59.53
R-7	3:7	1:21	13.09	156.06	60.11
R-8	3:8	1:24	13.06	157.38	60.58

**Table 2:** Experimental design for the conversion of antibiotic fermentation residue by BSFL under different ratios of larva number to feed.

Group	Ratio of Larva Number to Feed	Waste Weight (g)	Number of Larvae
1	1:1	200	200
2	1.2:1	200	240
3	1.5:1	200	300
4	1.8:1	200	360
5	2:1	200	400

**Table 3:** The larval yield and feed loss of BSFL reared on antibiotic fermentation residue and potato peel waste. Different lower-case letters indicate significant difference (*p* < 0.05).

Feeding Mixture	Fresh Larval Mass (g)	Dry Larval Mass (g)	Material Mass Reduction (g)
R-1	15.54 ± 0.24 ^e^	4.25 ± 0.05 ^d^	13.54 ± 0.12 ^f^
R-2	22.58 ± 0.4 ^d^	6.26 ± 0.04 ^bc^	15.35 ± 0.69 ^e^
R-3	24.78 ± 0.17 ^c^	6.5 ± 0.26 ^abc^	19.05 ± 0.15 ^d^
R-4	25.61 ± 0.62 ^bc^	6.16 ± 0.38 ^c^	18.93 ± 0.42 ^d^
R-5	26.45 ± 0.6 ^ab^	6.76 ± 0.21 ^a^	20.01 ± 0.72 ^cd^
R-6	26.89 ± 0.13 ^a^	6.69 ± 0.13 ^ab^	21.05 ± 0.39 ^bc^
R-7	27.08 ± 0.34 ^a^	6.65 ± 0.12 ^ab^	21.70 ± 0.75 ^ab^
R-8	26.77 ± 0.67 ^a^	6.82 ± 0.10 ^a^	22.41 ± 0.29 ^a^

**Table 4:** Nosiheptide spike recovery rate experiment.

Spiked Concentration (mg/kg)	Spike Recovery Rate (%)
200	109.61 ± 0.33
1000	97.21 ± 1.68

**Table 5:** The size of the inhibition zone of antibiotic residues before and after degradation. Different lower-case letters indicate significant difference (*p* < 0.05).

Treatment	High (mm)	Low (mm)
before transformation	3.87 + 0.60 ^a^	1.93 + 0.25 ^b^
after transformation	0 ^c^	0 ^c^

note: high group: antibiotic fermentation residue: potato peel waste = 1:6, low group: antibiotic fermentation residue: potato peel waste = 1:15.

**Table 6:** Conversion indicators of high- and low-concentration antibiotic fermentation residues treated by BSFL. Different lower-case letters indicate significant difference (*p* < 0.05).

Treatment (Antibiotic Fermentation Residue/Potato Peel Waste)	Total Fresh Weight (g)	Material Reduction Rate (%)	Bioconversion Rate (%)	Feed Conversion Rate (%)
High (1:6)	64.29 ± 0.82 ^b^	21.58 ± 1.00 ^b^	9.36 ± 0.45 ^b^	2.31 ± 0.01 ^a^
Low (1:15)	75.43 ± 1.46 ^a^	35.82 ± 1.89 ^a^	15.16 ± 0.77 ^a^	2.38 ± 0.25 ^a^

**Table 7:** Composition and nutritional profile of BSFL fed with high- and low-concentration antibiotic fermentation residues. Different lower-case letters indicate significant differences (*p* < 0.05).

	High (1:6)	Low (1:15)
Nutritional composition (% dry matter)		
Crude protein	35.64 ± 0.66 ^a^	30.02 ± 0.05 ^b^
Crude fat	25.03 ± 0.60 ^b^	32.65 ± 0.70 ^a^
Amino acid (g/100 g)		
Asp	1.96 ± 0.02	2.07 ± 0.05
Thr	0.95 ± 0.01	1.04 ± 0.02
Ser	1.07 ± 0.02	1.16 ± 0.03
Glu	3.03 ± 0.03	3.08 ± 0.07
Gly	1.41 ± 0.01	1.50 ± 0.03
Ala	1.90 ± 0.04	2.17 ± 0.05
Val	1.39 ± 0.02	1.50 ± 0.03
Met	0.16 ± 0.01	0.07 ± 0.00
Ile	0.62 ± 0.02	0.65 ± 0.00
Leu	1.09 ± 0.01	1.20 ± 0.01
Tyr	0.52 ± 0.01	0.91 ± 0.03
Phe	0.82 ± 0.03	0.77 ± 0.36
His	0.73 ± 0.03	0.52 ± 0.01
Lys	1.24 ± 0.06	0.98 ± 0.03
Arg	1.02 ± 0.02	1.02 ± 0.03
Pro	1.43 ± 0.02	1.53 ± 0.09
Total amino acids	19.33 ± 0.12	20.17 ± 0.39

**Table 8:** Fatty acid composition and content of BSFL fed with high- and low-concentrations of antibiotic fermentation residue.

Fatty Acid (%)	High (1:6)	Low (1:15)
Capric acid (10:0)	1.55	0.78
Lauric acid (12:0)	36.73	24.79
Myristate (14:0)	6.77	5.30
Palmitic acid (16:0)	13.34	14.62
Palmitoleic acid (16:1)	4.69	4.49
Stearic acid (18:0)	2.73	3.31
Oleic acid (18:1)	23.79	31.85
Linoleic acid (18:2)	7.53	11.20
Linolenic acid (18:3)	0.69	1.13
Arachidic acid (20:0)	0.25	0.35
Arachidonic acid (20:1)	0.08	0.12

## Data Availability

Data will be made available on request.
